# Beyond Black Boxes: Interpretable AI with Explainable Neural Networks (ENNs) for Acute Myocardial Infarction (AMI) Using Common Hematological Parameters

**DOI:** 10.3390/medicina61091552

**Published:** 2025-08-29

**Authors:** Zeynep Kucukakcali, Ipek Balikci Cicek

**Affiliations:** Department of Biostatistics and Medical Informatics, Faculty of Medicine, Inonu University, 44280 Malatya, Turkey; ipek.balikci@inonu.edu.tr

**Keywords:** acute myocardial infarction (AMI), Explainable Neural Network (ENN), SHAP, hematological biomarkers, machine learning, interpretability, precision medicine

## Abstract

*Background and Objectives*: This study aims to evaluate the diagnostic potential of routinely available hematological parameters for acute myocardial infarction (AMI) by employing an Explainable Neural Network (ENN) model that combines high predictive accuracy with interpretability. *Materials and Methods*: A publicly available dataset comprising 981 individuals (477 AMI patients and 504 controls) was analyzed. A broad set of hematological features—including white blood cell subtypes, red cell indices, and platelet-based markers—was used to train an ENN model. Bootstrap resampling was applied to enhance model generalizability. The model’s performance was assessed using standard classification metrics such as accuracy, sensitivity, specificity, F1-score, and Matthews Correlation Coefficient (MCC). SHapley Additive exPlanations (SHAP) were employed to provide both global and individualized insights into feature contributions. *Results*: The study analyzed hematological and biochemical parameters of 981 individuals. The explainable neural network (ENN) model demonstrated excellent diagnostic performance, achieving an accuracy of 94.1%, balanced accuracy of 94.2%, F1-score of 93.9%, and MCC of 0.883. The AUC was 0.96, confirming strong discriminative ability. SHAP-based explainability analyses highlighted neutrophils (NEU), white blood cells (WBC), RDW-CV, basophils (BA), and lymphocytes (LY) as the most influential predictors. Individual- and class-level SHAP evaluations revealed that inflammatory and erythrocyte-related parameters played decisive roles in AMI classification, while distributional analyses showed narrower parameter ranges in healthy individuals and greater heterogeneity among patients. *Conclusions*: The findings suggest that cost-effective, non-invasive blood parameters can be effectively utilized within interpretable AI frameworks to enhance AMI diagnosis. The integration of ENN with SHAP provides a dual benefit of diagnostic power and transparent rationale, facilitating clinician trust and real-world applicability. This scalable, explainable model offers a clinically viable decision-support tool aligned with the principles of precision medicine and ethical AI.

## 1. Introduction

Acute myocardial infarction (AMI), a critical condition marked by the sudden loss of blood flow to the heart muscle, is closely associated with various hematological parameters. These parameters not only reflect the body’s physiological responses but may also serve as vital indicators of the prognosis and severity of AMI [1,2].

One prominent hematological marker linked to AMI is the mean platelet volume (MPV), which serves as a surrogate measure for platelet activity. Elevated MPV has been identified as an independent risk factor for myocardial infarction, attributed to its role in thrombus formation and atherosclerosis [3,4,5]. Higher MPV signifies larger, more reactive platelets, contributing to the development of atherothrombotic events [5]. In the context of AMI, an increased MPV correlates with poorer responses to treatment modalities, such as thrombolysis, emphasizing its potential utility in risk stratification [6].

Another critical parameter is the neutrophil-to-lymphocyte ratio (NLR), which serves as a proxy for systemic inflammation. Studies indicate that elevated NLR is associated with increased short- and long-term mortality following AMI [7,8]. The inflammatory response, which is a hallmark of myocardial infarction, is reflected in leukocytosis commonly observed in these patients. This inflammatory state not only exacerbates myocardial damage but may also complicate recovery processes [9,10].

Moreover, the red blood cell distribution width (RDW) has emerged as a relevant prognostic marker in AMI. Elevated RDW levels have been linked to poor clinical outcomes, indicating potential underlying systemic inflammation and instability in erythropoiesis [11,12]. RDW acts as a predictor of mortality in patients suffering from myocardial infarction, accentuating its significance as a readily available biomarker in clinical practice [12]. The heterogeneity in erythrocyte volume it represents can highlight inflammatory processes critical to cardiovascular disease pathogenesis [13].

The interplay between hematological parameters and AMI is further underscored in patients with hematological malignancies. Conditions such as acute lymphoblastic leukemia have been shown to present unique challenges when accompanied by acute coronary syndromes (ACS), often exacerbated by complications like thrombocytopenia, which can significantly increase in-hospital mortality rates [14,15]. These findings illustrate how specific hematologic profiles can influence the likelihood of experiencing an AMI and impact the overall clinical management of such patients [16].

In summary, various hematological parameters, notably MPV, NLR, and RDW, furnish critical insights into the pathophysiology of AMI. They act as both biomarkers of disease severity and indicators of patient prognosis, highlighting the importance of integrating hematological assessments into routine clinical evaluation for those at risk of or presenting with AMI.

In light of the aforementioned studies, the relationship between hematological parameters and AMI has been extensively investigated. A wide array of research has demonstrated that these routinely available blood-based biomarkers such as mean platelet volume (MPV), neutrophil-to-lymphocyte ratio (NLR), and red blood cell distribution width (RDW) are not only reflective of the body’s physiological and inflammatory responses to myocardial injury, but also serve as independent predictors of disease severity, treatment response, and clinical outcomes. Their accessibility, cost-effectiveness, and prognostic relevance have rendered them valuable tools in the early risk stratification and long-term management of patients with AMI. These findings collectively highlight the clinical significance of hematological indices in the context of cardiovascular disease, and particularly in the acute and subacute phases of myocardial infarction.

However, despite the expanding body of research on machine learning (ML) applications in cardiovascular medicine, the integration of explainable artificial intelligence (XAI) approaches remains notably limited—particularly in studies aiming to model and interpret the role of hematological biomarkers in AMI. While ML algorithms have demonstrated impressive capabilities in classifying disease states, predicting outcomes, and identifying complex, non-linear relationships within biomedical data, their adoption in high-stakes clinical settings has been hampered by the “black box” nature of many models. Only a small subset of studies have prioritized transparency and interpretability by incorporating XAI frameworks, which are essential for ensuring clinician trust, regulatory compliance, and ethical implementation [17]. In the context of AMI, where timely and accurate decision-making can significantly affect patient outcomes, the ability to understand how individual hematological features contribute to model predictions is not merely desirable but necessary. Thus, there is a pressing need to bridge the gap between predictive power and interpretability by embedding XAI methodologies into cardiovascular ML research, particularly when leveraging routinely collected yet underutilized data such as complete blood count parameters.

In response to the need for interpretability in clinical ML applications, this study used the Neural Additive Model (NAM), an interpretable machine learning model from the Explainable Neural Network (ENN) family. ENNs are neural networks specifically designed to maintain explainability by imposing architectural constraints and utilizing visualization techniques that reveal internal representations. These models preserve much of the flexibility associated with deep learning while ensuring that input features are transparently mapped to outputs. By balancing performance with explainability, ENNs help clinicians understand the logic behind predictions, increase confidence, and enable more informed decision-making in complex clinical scenarios [18].

By applying these XAI-based approaches to routinely collected hematological parameters, this study aims to provide both accurate prediction and human-understandable explanations of AMI outcomes. This focus is essential for translating ML advances into actionable insights in cardiovascular care, particularly where early detection and risk stratification based on accessible blood-based biomarkers remain a clinical priority.

## 2. Materials and Methods

### 2.1. Dataset

In this study, we utilized an openly accessible dataset titled “Heart disease,” available on Mendeley Data (V1, https://doi.org/10.17632/m482gb564t.1). The dataset, provided under a Creative Commons Attribution-NonCommercial-NoDerivatives 4.0 License, includes routinely collected hematological and biochemical parameters from a total of 981 individuals—comprising 477 patients diagnosed with AMI and 504 controls. In the original dataset, patients in the AMI group were those admitted to the hospital due to a confirmed diagnosis of AMI. Therefore, it is highly likely that hematological measurements were obtained after diagnosis confirmation; however, the dataset does not provide precise timestamps relative to the time of diagnosis, and this temporal relationship cannot be definitively determined. This limitation has been explicitly stated in the revised manuscript, and we recommend that future prospective studies record exact sampling times to enable temporal alignment and ensure robust early-detection modelling.

The dataset encompasses a structured set of clinical variables relevant to cardiovascular diagnostics and consists of the following:

Demographic variables: age, gender (0: female, 1: male)
Hematological parameters: white blood cell (WBC), red blood cell (RBC), hemoglobin (HGB), hematocrit (HCT), mean corpuscular volume (MCV), mean corpuscular hemoglobin (MCH), mean corpuscular hemoglobin concentration (MCHC), red cell distribution width—standard deviation (RDW-SD), red cell distribution width—coefficient of variation (RDW-CV), platelet count (PLT), mean platelet volume (MPV), platelet distribution width (PDW), procalcitonin (PCT), basophil (BA), eosinophil (EO), lymphocyte (LY), monocyte (MO), neutrophil (NEU)
Derived indices: neutrophil-to-lymphocyte ratio (NEU/LY), platelet-to-lymphocyte ratio (PLT/LY), mean platelet volume-to-lymphocyte ratio (MPV/LY), lymphocyte-to-monocyte ratio (LY/MO)
Target variable: label (0 = control, 1 = AMI)

The decision to reuse this dataset in our current study was guided by several factors. First, its public availability ensures research transparency and reproducibility. Second, the hematological parameters incorporated in this study are not only cost-effective and routinely obtainable in most clinical settings but also increasingly acknowledged in the literature for their substantial diagnostic and prognostic relevance in AMI. Their accessibility and growing evidence base make them particularly well-suited for inclusion in interpretable machine learning frameworks aimed at real-world clinical application.

By employing this comprehensive and validated dataset, our study aims to extend the findings of prior work through the application of an interpretable machine learning model. Specifically, we explore the diagnostic utility of explainable models that the ENN uses, with the goal of improving clinical decision support systems based on routine blood test results.

### 2.2. Explainable Artificial Intelligence (XAI) and Neural Network (ENN)

Explainable Artificial Intelligence (XAI) and ENN are pivotal areas of research aimed at enhancing the interpretability and transparency of AI systems, particularly complex models like neural networks. XAI seeks to provide insights into the decision-making processes of AI models, addressing the critical need for accountability and comprehension in AI applications across various sectors.

XAI integrates a diverse array of methodologies aimed at making machine learning models more transparent, particularly when dealing with highly complex architectures such as deep neural networks. Unlike traditional models like linear regression or decision trees, which are inherently interpretable, neural networks often function as “black boxes” due to their multilayered, nonlinear structure. Somani et al. [19] highlight how gradient-based interpretation techniques have contributed significantly to bridging the gap between high predictive performance and the need for model transparency. However, these post-hoc methods, while valuable, may not always faithfully represent the internal reasoning of the model. As a response to this limitation, intrinsically interpretable approaches—such as ENNs have been proposed. ENNs are designed to embed explainability directly into the architecture itself, enabling simultaneous optimization of predictive accuracy and human interpretability. By imposing architectural constraints and using attention or rule-based mechanisms, ENNs facilitate real-time, transparent decision-making and foster greater trust in model outputs, especially in high-stakes domains such as healthcare and finance [20].

Unlike traditional black-box models, which rely on external tools to approximate the rationale behind predictions, ENNs are designed so that each layer, weight, or component carries a clear semantic role [21]. These models commonly use structural constraints such as sparse connectivity, modular decomposition, or rule-based logic to ensure that decision pathways can be transparently traced and understood. ENNs encompass a broad family of architectures, including concept bottleneck models, prototype-based networks, and self-explaining neural networks—all of which aim to balance predictive performance with human-understandable reasoning [21]. Their design allows for real-time interpretability, which is particularly valuable in domains like healthcare and criminal justice, where decisions must be explainable and ethically sound.

The Neural Additive Model (NAM) is an interpretable neural network approach [22]. This model is a non-linear prediction method based on independent processing and contribution calculation of each feature. NAM employs separate neural networks for each feature, enhancing model interpretability while simultaneously capturing complex relationships. Its fundamental architecture incorporates distinct neural networks for each feature, with their outputs aggregated through a global summation function. This approach, unlike traditional black-box models, allows for a detailed understanding of each feature’s contribution to the classification outcome [23].

### 2.3. Modelling

In this study, we used a Neural Additive Model (NAM) to provide an interpretable and precise approach for classifying blood parameters. The proposed model integrates advanced machine learning principles with a multi-layered neural network architecture to enhance both predictive performance and feature-level interpretability.

The NAM architecture processes each input feature (blood parameter) through a dedicated feature-specific subnetwork, allowing for unique transformations and enabling granular contribution analysis. Each subnetwork consists of two hidden layers with ReLU activation functions, followed by dropout regularization (rate = 0.2). Feature-specific outputs are aggregated through an additive framework, and the combined contributions are passed through a global sigmoid activation function to generate the final prediction. This design facilitates not only accurate classification but also transparent interpretation of the relative importance and directional impact of each parameter.

The dataset was reviewed for missing data and extreme outliers. There were no missing values, and no extreme outliers were detected beyond physiologically plausible ranges. The dataset was partitioned using an 80/20 stratified train–test split to preserve balanced class distributions between healthy and diseased samples. Prior to training, all features were standardized via Z-score normalization, with the scaler fitted exclusively on the training set to prevent information leakage.

Hyperparameter optimization was conducted through systematic evaluation of multiple parameter configurations: Architecture exploration involved testing various hidden layer sizes ([32, 16], [64, 32], [128, 64]) to balance model expressivity with computational efficiency. Learning rate analysis evaluated different rates (0.0001, 0.001, 0.01) for optimal convergence characteristics, while regularization tuning compared dropout rates (0.1, 0.2, 0.3) and weight decay values for generalization performance. The final configuration ([64, 32] hidden units, lr = 0.001, dropout = 0.2) was selected based on superior validation performance and consistent training dynamics. The optimized NAM was trained using the Adam optimizer (learning rate = 0.001, L2 weight decay = 1 × 10^−5^) with Binary Cross-Entropy Loss as the optimization criterion. The training process was conducted for a maximum of 150 epochs, with early stopping (patience = 20) triggered by test set accuracy, and a ReduceLROnPlateau scheduler (patience = 10, factor = 0.5) to dynamically adjust the learning rate. To mitigate overfitting, dropout regularization (rate = 0.2) was applied across hidden layers, and training convergence was monitored via loss curves, with early stopping activated at epoch 45 to achieve an optimal balance between accuracy and interpretability. For performance evaluation, multiple metrics were employed, including accuracy, balanced accuracy, Matthews Correlation Coefficient, precision, recall, F1-score, and ROC AUC. These metrics were calculated exclusively on the 20% hold-out test set obtained from the stratified 80/20 split. Bootstrap resampling with 1000 iterations was then applied to the test set results to calculate confidence intervals and assess the statistical significance of feature contributions.

All computational experiments were implemented in Python 3.11 using PyTorch for deep learning, with NumPy, SciPy, and scikit-learn for data processing and statistical analysis. Experiments were conducted on a standard desktop computer with a multi-core CPU and sufficient RAM for batch processing.

**Figure 1:**
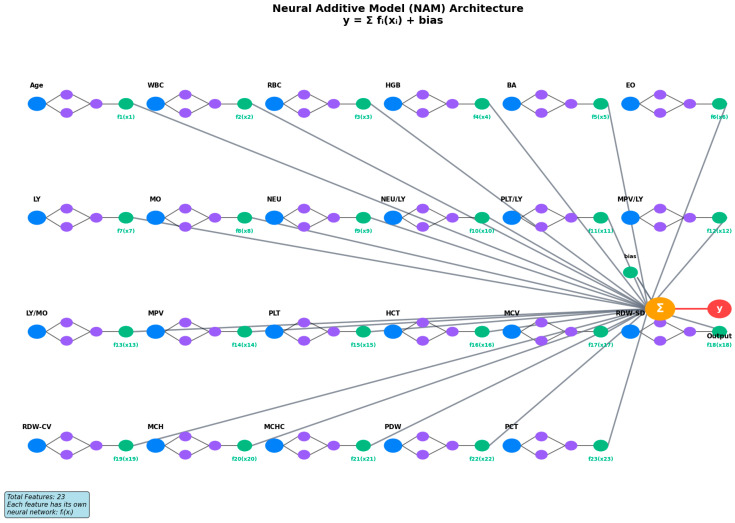
Neural Additive Model (NAM) architecture for blood parameter analysis. The model processes 23 features (Age, WBC, RBC, HGB, BA, EO, LY, MO, NEU, NEU/LY, PLT/LY, MPV/LY, LY/MO, MPV, PLT, HCT, MCV, RDW-SD, RDW-CV, MCH, MCHC, PDW, PCT) through individual neural networks fᵢ(xᵢ), enabling full explainability via additive structure: y = Σ fᵢ(xᵢ) + bias.

Figure 1 illustrates the proposed NAM architecture, where each of the 23 parameters undergoes independent neural network processing through dedicated feature networks fᵢ(xᵢ). This additive structure ensures complete explainability, as the final prediction y is computed as the linear sum of individual feature contributions plus a global bias term: y = Σᵢ₌_1_^23^ fᵢ(xᵢ) + bias.

## 3. Results

### 3.1. Modeling for AMI and Control Groups

This study was conducted using a dataset containing hematological and biochemical parameters of 981 individuals. The data consisted of 477 patients diagnosed with AMI and 504 control subjects. To enhance statistical power and improve model training stability, bootstrap-based data augmentation was implemented, doubling the dataset size from 981 to 1962 samples. This approach involved generating an additional bootstrap sample of equal size through resampling with replacement from the original dataset, then concatenating it with the original data to create an expanded training corpus while preserving the underlying data distribution characteristics. The ages of the participants ranged from 20 to 82 years, with an average age of 59.17 (±10.851). The gender distribution was 42.1% female (n = 413) and 57.9% male (n = 568). The baseline hematological parameters of patients with AMI and controls are presented in Table 1.

Significant hematological changes were observed in the AMI group compared to the control group. Significant increases were detected in inflammatory markers, including WBC (7.5 vs. 10.38, *p* < 0.001), neutrophil count (4.3 vs. 6.45, *p* < 0.001), and NLR (1.89 vs. 2.697, *p* < 0.001), reflecting the acute inflammatory response developing in AMI. Among platelet function parameters, a significant decrease in MPV (10.1 vs. 9.4 fL, *p* < 0.001) and an increase in PDW (12 vs. 15.9 fL, *p* < 0.001) were observed, indicating increased platelet activation and consumption. Among erythrocyte indices, a slight decrease in MCV (87.2 vs. 86.3 fL, *p* = 0.003) and an increase in RDW-CV (13.2 vs. 13.6, *p* < 0.001) were evaluated as indicators of hematopoietic stress associated with the acute condition. These changes in hematological parameters can be used as supportive findings in the diagnosis of AMI.

The developed explainable artificial intelligence model ENN demonstrated high classification performance in AMI diagnosis. The diagnostic performance of the model was evaluated using basic classification metrics such as sensitivity, specificity, accuracy, F1-score, and Matthews Correlation Coefficient (MCC). According to the results obtained, the overall accuracy of the model was 94.1% (95% confidence interval: 0.918–0.965), the balanced accuracy was 94.2% (95% CI: 0.919–0.965), and the F1-score was 93.9% (95% CI: 0.915–0.963). Additionally, the MCC value was 0.883 (95% CI: 0.852–0.915), indicating that the model can effectively distinguish between the two classes. A detailed summary of the performance metrics is presented in Table 2.

**Figure 2:**
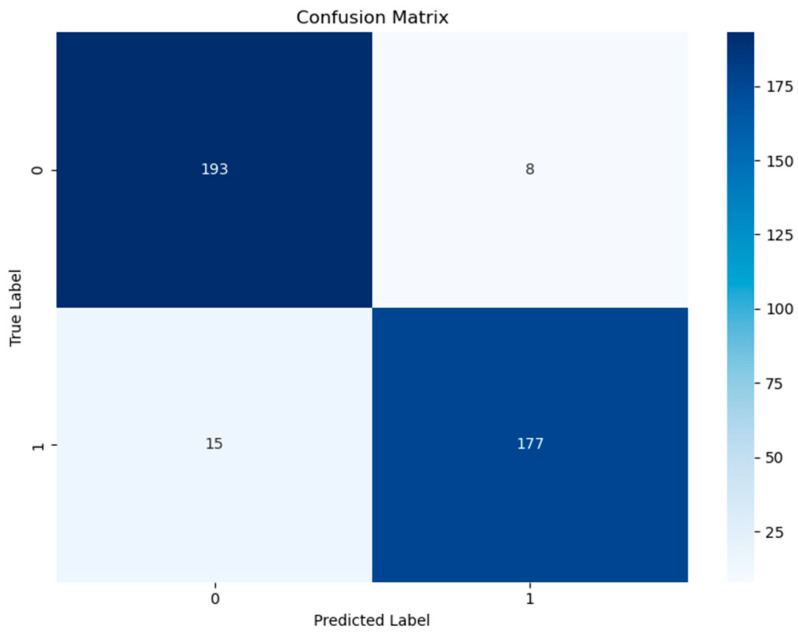
Confusion matrix of the model.

**Figure 3:**
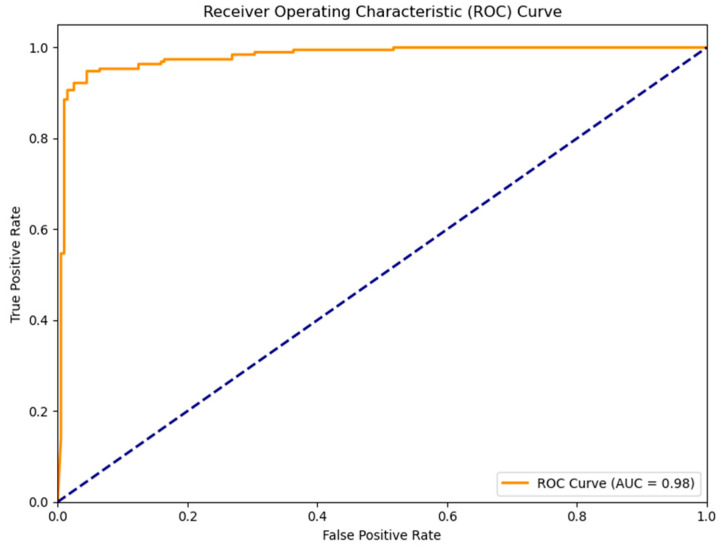
ROC curve and area under the curve (AUC) analysis.

The confusion matrix presented in Figure 2 clearly shows that the model can correctly classify real patients and control individuals. The high number of true positives and true negatives observed in the matrix supports the classification stability of the model. Additionally, the ROC curve (Receiver Operating Characteristic Curve) shown in Figure 3 illustrates the sensitivity and specificity levels of the model under different threshold values. The area under the ROC curve (AUC) value is calculated as 0.96, indicating that the model has a very high diagnostic discriminative ability.

**Figure 4:**
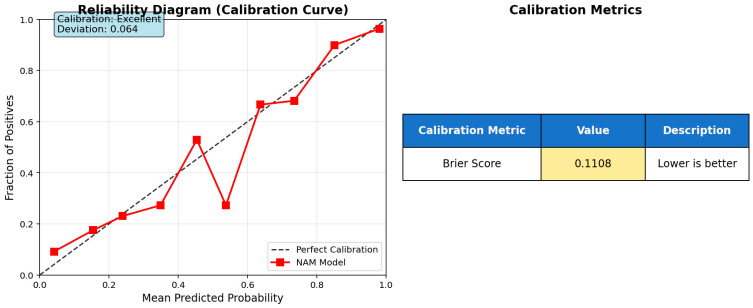
Calibration analysis with reliability diagram and Brier score.

The calibration curve for the model is shown in Figure 4.

To evaluate the model’s reliability for clinical risk estimation, a comprehensive calibration analysis was conducted on the test dataset. The reliability diagram (Figure 4) demonstrates that the NAM model predictions exhibit good alignment with the perfect calibration line, achieving a calibration deviation of 0.064. This low deviation indicates satisfactory agreement between predicted probabilities and actual outcomes across different probability ranges.

The Brier score of 0.1108 confirms acceptable probabilistic prediction accuracy for clinical applications, falling within the established threshold for medical risk assessment tools (<0.15). This calibration performance demonstrates that the model produces reasonably well-calibrated probability estimates, making it suitable for clinical decision support where reliable risk quantification is required.

The calibration results, combined with the model’s inherent explainability through additive feature contributions, support the potential deployment of this approach in clinical settings for blood parameter-based risk stratification.

**Figure 5:**
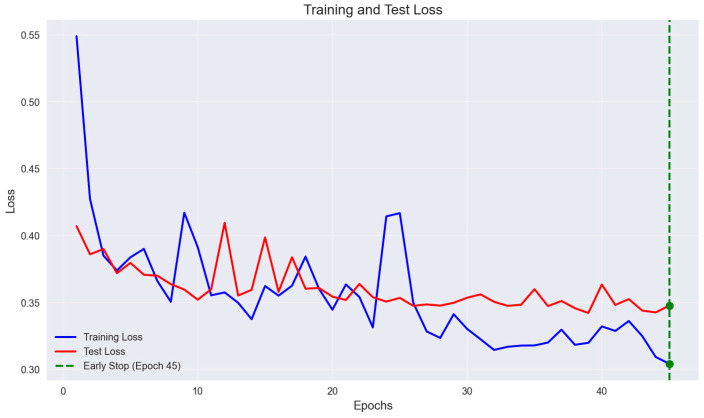
Training and test loss curves demonstrating model convergence.

The training dynamics of the ENN, illustrating the progression of both training and test loss across epochs and the point of early stopping, are presented in Figure 5.

Training dynamics of the ENN model, showing the progression of training loss (blue) and validation/test loss (red) across epochs. The early stopping criterion was triggered at epoch 45 (green dashed line). Importantly, the model weights from epoch 39, where the lowest validation loss was observed, were restored and used for the final evaluation, rather than the weights from the last epoch before stopping.

**Figure 6:**
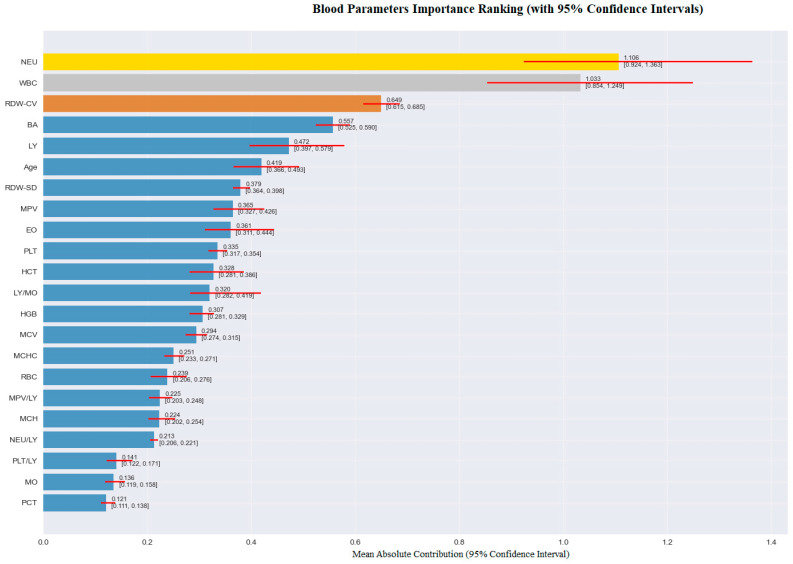
Feature importance analysis of hematological parameters contributing to model explainability. Colored bars represent mean absolute SHAP values across all samples, with red horizontal lines indicating 95% confidence intervals derived from bootstrap resampling (n = 1000 iterations). Values in parentheses show the confidence interval bounds for each parameter. Features are ranked in descending order by mean importance magnitude.

An importance analysis of blood parameters was performed to increase the explainability of the model and to determine which variables contributed to the classification decisions. The findings obtained within this scope are visualized in Figure 6. According to the analysis results presented in Figure 3, the parameter with the highest contribution to the classification decision was NEU. NEU was followed by WBC, RDW-CV, BA, and LY, respectively. These findings indicate that hematological parameters associated with inflammation play a decisive role in the diagnosis of AMI.

**Figure 7:**
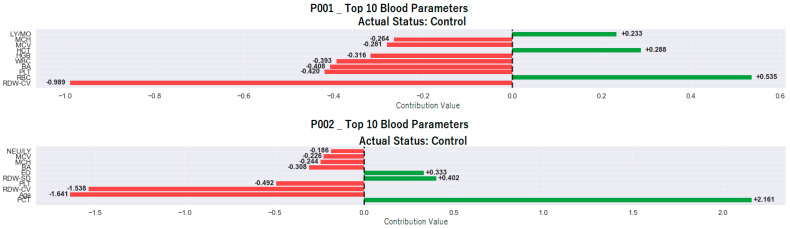
SHAP analysis of two individuals (P001 and P002) correctly classified as control by the model.

Figure 7 presents the SHAP analyses performed for two individuals (P001 and P002) classified as control by the model. This analysis provides a detailed overview of which hematological variables contributed positively (in favor of the patient) or negatively (in favor of the control individual) to the classification decision for each individual.

In the P001 individual, negative contributions dominate the classification decision and have been decisive in directing the individual toward the “control” class. The highest negative contribution is −0.989, associated with the RDW-CV parameter. This is followed by PLT with −0.420, BA with −0.408, WBC with −0.393, HGB with −0.316, MCV with −0.281, and MCH with −0.264. The fact that these variables are within normal limits or close to the control individual profile has shaped the model’s decision in a negative direction.

Positive contributions were limited, with the highest positive contribution coming from the RBC parameter at +0.535. HCT at +0.288 and LY/MO at +0.233 also contributed to a limited extent. This pattern indicates that the classification decision was largely based on negative contributions.

In the P002 individual, a more balanced structure was observed, where both negative and positive contributions were effective in the classification decision. Among the negative contributions, the age parameter stands out, providing a high negative contribution of −1.641. This is followed by RDW-CV with −1.538, PLT with −0.492, BA with −0.308, MCH with −0.244, MCV with −0.226, and NEU/LY ratio with −0.186. These values indicate that the individual’s hematological profile is closer to the control class and that the model, therefore, gave more weight to negative contributions.

On the other hand, it is noteworthy that positive contributions are also relatively strong in this individual. In particular, the PCT parameter, with +2.161, made a significant positive contribution to the model’s decision. Additionally, RDW-SD with +0.402 and EO with +0.333 also contributed positively to the classification decision. However, these positive contributions were balanced by the total negative contributions and did not prevent the model from classifying the individual as healthy.

When the SHAP analyses of both individuals are evaluated together, it is observed that the model’s decisions regarding the control class are based on the negative contributions of variables such as RDW-CV, age, PLT, BA, MCH, and MCV. Despite the presence of positive contributions, the fact that they did not change the direction of the decision shows that the model can reliably interpret multidimensional contribution patterns and evaluate the individual’s overall hematological profile in a comprehensive manner that would assign them to the control class.

The colors used in the SHAP graphs represent the value level of the relevant variable in the individual. In this context, red tones indicate high values of the variable, while green tones indicate low values. Thanks to this color coding, not only can the effect of a single variable on the classification decision be visually tracked, but also the value level at which this effect occurs. Thus, the model’s decision-making process can be transparently evaluated based on the direction and intensity of the contribution, as well as the magnitude of the observation.

**Figure 8:**
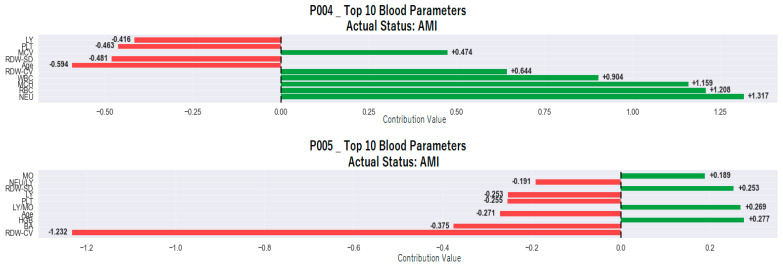
SHAP analysis of two individuals (P004 and P005) classified as AMI cases by the model.

Figure 8 presents the SHAP analysis results for two individuals (P004 and P005) classified as “patient” by the model based on an AMI diagnosis. These analyses quantitatively reveal which hematological variables contributed positively (in favor of the patient class) or negatively (in favor of the control class) to the model’s individual decision-making process. Different combinations of variables were found to influence the model’s decision in both patients, indicating that AMI has a heterogeneous biological profile at the individual level.

Positive contributions were dominant among the factors shaping the classification decision in the P004 individual and were decisive in the model’s assignment of the individual to the patient class. The highest positive contribution is +1.317, belonging to the NEU parameter. This value is followed by +1.208 for RBC, +1.159 for MCH, +0.904 for WBC, +0.644 for RDW-CV, and +0.474 for MCV. These values reveal that inflammation and erythrocyte parameters in the individual’s hematological profile are perceived by the model as indicators with a high correlation with AMI diagnosis.

Negative contributions remained limited but were partially effective in the decision. In particular, negative contributions were observed for variables such as age with −0.594, RDW-SD with −0.481, PLT with −0.463, and LY with −0.416. These contributions were insufficient to alter the classification decision, and the individual was assigned to the “patient” class due to the dominance of positive contributions.

In individual P005, negative contributions are clearly dominant, and a different pattern emerges in the model’s decision-making mechanism. The highest negative contribution comes from the RDW-CV parameter at −1.232. This was followed by BA with −0.375, age with −0.271, PLT with −0.255, LY with −0.253, and NEU/LY ratio with −0.191. The fact that these parameters show values close to low or healthy profiles indicates that the individual exhibits characteristics similar to control individuals in some aspects.

However, the parameters contributing positively to the classification decision remained at lower levels. The highest positive contribution was +0.277 for the HGB parameter, followed by +0.269 for the LY/MO ratio, +0.253 for RDW-SD, and +0.189 for MO.

These positive contributions are limited in scope, and their impact on the decision is not as pronounced as that of the negative contributions. The color scale used in the SHAP analysis reflects the value level of each variable in the individual. In this context, red tones represent high values of the variable, while blue tones represent low values.

Thus, in addition to the positive or negative contribution, the color coding also visualizes the level of observation associated with this contribution. This feature clearly reflects that the model relates its decisions not only to the magnitude of the contribution but also to the magnitude of the parameter values.

**Figure 9:**
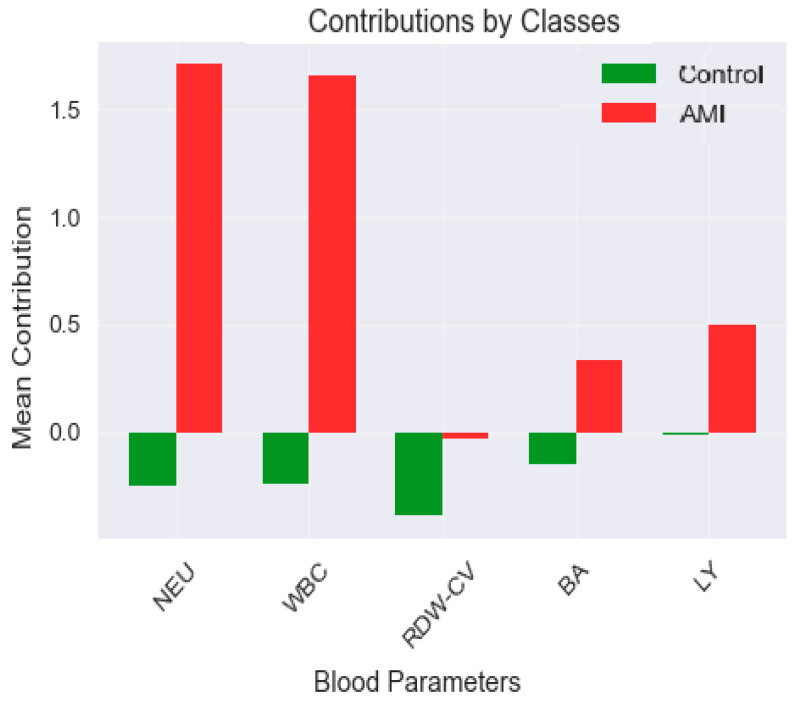
Comparative SHAP analysis of hematological features by class (AMI vs. control).

Figure 9 presents a comparative analysis of the contributions of hematological parameters used in the model to classification decisions based on class (patient and control individuals). This analysis is of great importance in terms of visualizing global contribution patterns, which enable the understanding of group-level decision mechanisms in explainable artificial intelligence models. According to the model output, the top five variables contributing the highest average contribution to the classification decision were NEU, WBC, RDW-CV, BA, and LY. These variables stand out with their distinctive contribution profiles, particularly between patients and control individuals.

In the patient group, the NEU, WBC, and RDW-CV parameters were observed to contribute positively to the classification decision. This finding indicates that the values of these inflammation-related parameters are significantly different in individuals diagnosed with AME and that the model associates these deviations with the patient class. Similarly, immune system components such as BA and LY were also found to contribute positively to the classification decision in the patient group. These contribution patterns indicate that the model has developed sensitivity to parameters associated with inflammatory processes.

On the other hand, the contribution levels of these variables are significantly lower in the control group. In control individuals, the model considered these variables less influential in the classification decision because they fall within the normal reference range. In this group, the contributions of some variables were negative and supported labeling the individual as healthy. In particular, the low contribution values of parameters such as RDW-CV, LY, and BA supported the model’s decisions in the control group.

In conclusion, the comparative analysis presented in Figure 6 clearly shows how the model weights variables in different classes. The fact that parameters such as NEU, WBC, and RDW-CV become particularly decisive in the patient group supports the biological importance of these variables in AMI diagnosis and highlights the contribution of the explainable artificial intelligence approach to clinical interpretability.

**Figure 10:**
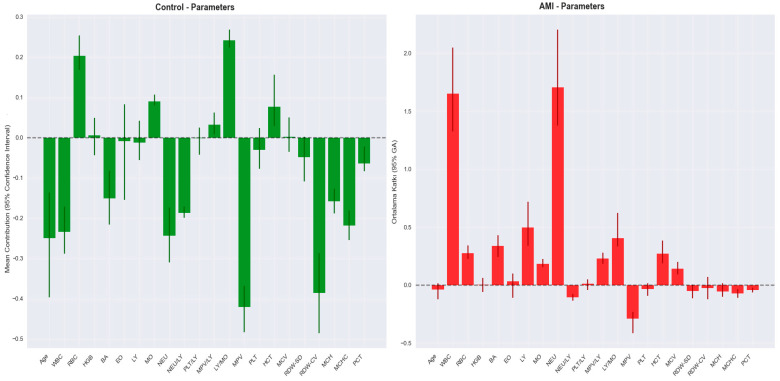
Class-wise distribution of hematological features in AMI and control groups.

Figure 10 compares the distributions of hematological parameters used in the model in patients and control individuals, visualizing the variations between classes. This analysis provides important clues for understanding which parametric distribution differences the model is sensitive to and on which structural variations the decision mechanism is based.

The visual analysis revealed that parameter distributions in control individuals were observed to be narrower and more homogeneous. This indicates that the model recognizes examples belonging to the control class through a more distinct pattern. In particular, variables such as RDW-CV, WBC, NEU, and age showed tighter distributions in the control group.

On the other hand, the distributions of these parameters in the patient group exhibited a significantly broader and more heterogeneous structure. The variation in inflammatory markers such as NEU and WBC reflects the biological diversity in the patient group, while the variability in parameters such as RDW-CV and age enhances the model’s ability to adapt to different patient profiles. These distributional differences also explain the underlying cause of the individual contribution diversity observed in SHAP analyses.

Overall, the distribution analyses presented in Figure 9 reveal that the model’s high diagnostic power is not only based on average values but also on parameter variations and that it has a flexible classification structure that takes into account inter-individual differences.

### 3.2. Comparative Performance Analysis of ENN and Alternative Classifiers for AMI and Control Groups

In this section, to provide a robust and transparent context for interpreting the diagnostic performance of the proposed Explainable Neural Network (ENN), we conducted a systematic benchmarking study against three well-established and widely adopted machine learning classifiers—Random Forest (RF), Support Vector Machine (SVM), and Extreme Gradient Boosting (XGBoost). All models were evaluated under identical experimental conditions to ensure methodological fairness, employing a stratified 80/20 train–test split to preserve class balance. Bootstrap resampling with 1000 iterations was applied to each model’s test set results to calculate 95% confidence intervals and assess the statistical significance of performance differences, consistent with the methodology used for ENN feature analysis. All models were evaluated under identical experimental conditions to ensure methodological fairness, applying Z-score normalization to all continuous features to standardize input scales across all splits.

**Figure 11:**
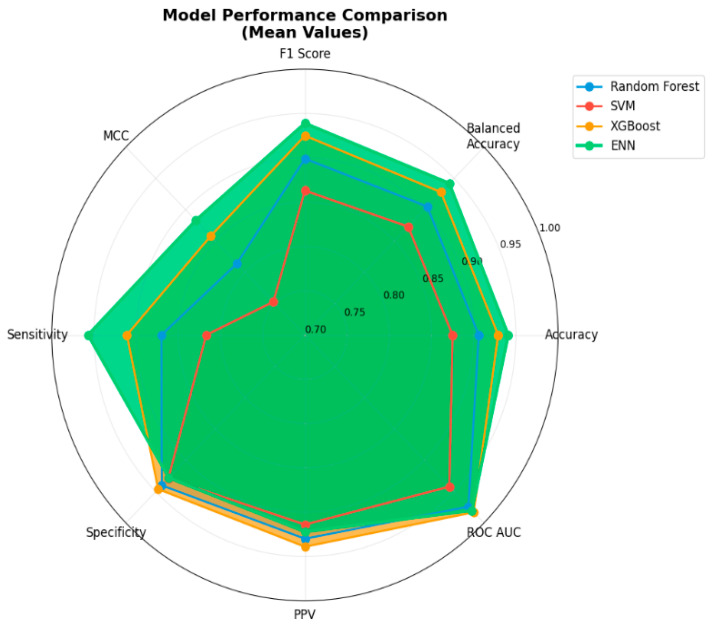
Radar chart analysis of model performance across key metrics.

The hyperparameters for all baseline models (RF, SVM, and XGBoost) were systematically optimized using grid search with 5-fold stratified cross-validation on the training set. We searched across: Random Forest (n_estimators [50, 80, 100, 120], max_depth [8, 10, 12], min_samples_split [5, 10, 15]), SVM (C [0.1, 1, 10], gamma [0.001, 0.01, 0.1, ‘scale’]), and XGBoost (learning_rate [0.05, 0.08, 0.1], max_depth [3, 5, 7], and subsample [0.7, 0.8, 0.9]). The final hyperparameter combination was selected based on the highest mean cross-validated balanced accuracy. The resulting optimized configurations were: the RF model was implemented with 80 estimators, a maximum tree depth of 10, minimum samples per split of 10, minimum samples per leaf of 5, square-root feature subsampling, bootstrap aggregation, and out-of-bag scoring for internal validation; the SVM employed a radial basis function (RBF) kernel with C = 1 and probability estimation enabled, operating on standardized feature inputs to ensure optimal margin scaling; and the XGBoost classifier was tuned with 100 estimators, a maximum depth of 5, learning rate of 0.08, subsample and column sampling rates of 0.8, L1 regularization (α) set to 0.1, L2 regularization (λ) set to 0.5, minimum child weight of 5, and a gamma value of 0.1 to control tree complexity. This rigorous and harmonized benchmarking framework not only ensures a fair, one-to-one comparison with the ENN but also provides robust estimates of model performance with appropriate uncertainty quantification by minimizing variability introduced by preprocessing disparities or suboptimal hyperparameter configurations. The comparative results with bootstrap-derived confidence intervals are presented in Table 3. To provide a comprehensive visual comparison of model performance across all key metrics, Figure 11 presents a radar chart analysis that illustrates the relative strengths and weaknesses of each classifier. The radar visualization allows for simultaneous assessment of multiple performance dimensions, clearly demonstrating the performance profiles of Random Forest, SVM, XGBoost, and ENN across eight critical classification metrics.

According to Table 3, when considering 95% bootstrap confidence intervals derived from 1000 iterations across multiple data partitions, the performance landscape reveals important nuances that single-split evaluations might obscure. Ensemble methods—particularly XGBoost—emerged as strong black-box baselines, with XGBoost achieving competitive performance across all metrics. Random Forest also demonstrated solid performance, while SVM consistently lagged behind in most metrics, particularly sensitivity (0.817, 95% CI: 0.776–0.858) and MCC (0.753, 95% CI: 0.707–0.800).

The ENN model achieved numerically superior mean performance across most metrics, delivering the highest accuracy (0.941), balanced accuracy (0.942), F1-score (0.939), MCC (0.883), sensitivity (0.957), and NPV (0.960). However, bootstrap confidence interval analysis reveals critical insights into the statistical significance of these differences: the confidence intervals between ENN and XGBoost show overlapping ranges for several key metrics, including accuracy (ENN: 0.913–0.969 vs. XGBoost: 0.902–0.957), balanced accuracy, F1-score, and ROC-AUC, indicating that while ENN demonstrates numerically superior performance, the differences are not statistically significant for these metrics. This finding aligns with the expectation that both advanced models would perform comparably on this classification task.

Notably, ENN demonstrated statistically superior performance compared to SVM across all evaluated metrics (non-overlapping confidence intervals) and showed significant advantages over Random Forest in clinically critical metrics, including sensitivity (ENN: 0.916–0.998 vs. RF: 0.829–0.911) and NPV (ENN: 0.925–0.995 vs. RF: 0.852–0.922). These findings suggest that while ensemble methods like XGBoost remain competitive in terms of raw predictive performance, traditional individual classifiers fall short of the performance levels achieved by both ENN and XGBoost.

Crucially, beyond the comparable predictive performance with XGBoost, the ENN provides a fundamental clinical advantage through its inherent interpretability. Unlike black-box ensemble methods, the ENN’s intrinsically interpretable NAM-based architecture integrates SHAP analysis to deliver detailed, patient-level explanations alongside its predictions—a critical feature absent in XGBoost and other ensemble approaches. This interpretability-performance balance positions ENN as a valuable alternative for clinical applications, where model transparency is essential for fostering clinical trust, supporting evidence-based physician decision-making, and ensuring safe integration into routine clinical workflows. In healthcare contexts where regulatory requirements and clinical acceptance demand explainable AI solutions, the ENN’s combination of competitive performance with full interpretability represents a significant advantage over purely performance-oriented black-box approaches, making it the preferred choice for AMI diagnostic applications despite statistically comparable accuracy metrics with XGBoost.

Additionally, the chart displays mean performance values for Random Forest (blue), SVM (red), XGBoost (orange), and ENN (green) across eight metrics: accuracy, balanced accuracy, F1-score, MCC, sensitivity, specificity, PPV, and ROC AUC. The outer edge represents perfect performance (1.0), while the center represents the minimum scale value (0.7). The ENN (green area) demonstrates consistently high performance across most metrics, with the XGBoost (orange line) showing comparable performance levels. The SVM (red line) exhibits the most constrained performance profile, particularly in sensitivity and MCC, while Random Forest (blue line) shows intermediate performance across all metrics. The visualization emphasizes ENN’s balanced performance profile while highlighting the performance gaps between different model approaches.

### 3.3. Modeling for STEMI and NSTEMI Groups

Of the patients with AMI, 182 (38%) had a STEMI (ST-segment elevation MI), and 295 (62%) had a NSTEMI (non-ST-segment elevation MI). The AMI group does not include unstable angina. We repeated the modeling for STEMI and NSTEMI using the modeling procedures applied for the AMI and control groups. The results of the modeling are presented in Table 4.

**Figure 12:**
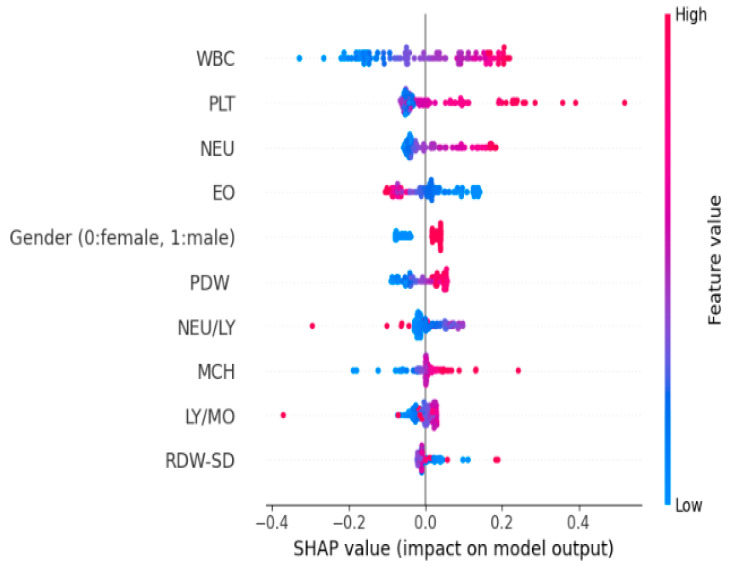
SHAP graph for STEMI and NSTEMI.

**Figure 13:**
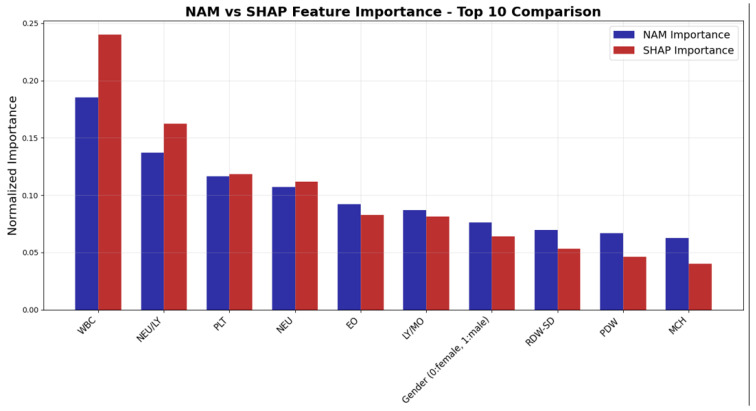
NAM vs. SHAP feature importance comparison.

The SHAP graph obtained from the modeling results is shown in Figure 12. Table 5 shows the variable importance values obtained from NAM and the variable importance values obtained from SHAP, while Figure 13 presents these values graphically.

This SHAP analysis demonstrates the strong discriminatory effect of WBC in STEMI and NSTEMI analysis. High WBC values (red dots, positive SHAP) indicate STEMI diagnosis, while platelets and neutrophils exhibit moderate discriminatory power. Ratios such as NEU/LY and LY/MO contribute modestly, whereas other hematological indices, including RDW-SD and MCH, exert minimal influence. These findings suggest that STEMI elicits a more severe inflammatory response compared to NSTEMI, underscoring the critical role of hematological markers in these distinctions.

This comparison plot reveals the concordance and differences between NAM’s intrinsic feature importance and SHAP’s model-agnostic explanations for the top 10 blood parameters. Both methodologies demonstrate strong agreement in identifying WBC as the most critical diagnostic marker, though SHAP assigns higher importance (0.240 vs. 0.188), reflecting its sensitivity to feature interactions and non-linear effects. The NEU/LY ratio similarly shows elevated SHAP importance, suggesting that this inflammatory marker’s complex relationships are better captured through perturbation-based explanations. Notably, both methods achieve identical ranking orders across all features, validating the robustness of the feature hierarchy. The convergence between NAM’s architectural importance and SHAP’s post-hoc explanations strengthens confidence in the clinical relevance of these hematological biomarkers for diagnostic classification.

## 4. Discussion

AMI remains one of the leading causes of morbidity and mortality worldwide, largely driven by the rupture of atherosclerotic plaques and the formation of occlusive thrombi within coronary arteries [24]. While clinical diagnosis traditionally relies on electrocardiographic changes and the detection of cardiac biomarkers such as troponins, recent research has increasingly focused on the role of hematological parameters in supporting early diagnosis and prognostication [24,25]. Blood-derived biomarkers such as white blood cell subtypes, platelet indices (including mean platelet volume and platelet distribution width), and red cell distribution width not only reflect systemic inflammatory and thrombotic states but also provide valuable insights into the underlying pathophysiology of AMI. Elevated platelet volume indices have been linked to worse outcomes following AMI, serving as independent predictors of mortality and poor perfusion quality after percutaneous coronary intervention (PCI) [26]. Similarly, higher red cell distribution width has been repeatedly associated with inflammation and adverse cardiovascular events, including AMI outcomes [27]. Additionally, total and differential white blood cell counts are well-recognized markers of systemic inflammation and have been shown to predict mortality or recurrent MI in cardiovascular patients [28]. These markers are inexpensive, widely accessible, and routinely measured in clinical practice, making them ideal candidates for integration into explainable machine learning models that aim to enhance diagnostic accuracy while preserving interpretability. The present study builds on this premise by evaluating the diagnostic value of routine hematological parameters in AMI using an inherently interpretable modeling approach.

These markers are inexpensive, widely accessible, and routinely measured in clinical practice, making them ideal candidates for integration into explainable machine learning models that aim to enhance diagnostic accuracy while preserving interpretability. Their cost-efficiency enables broad applicability in both high- and low-resource settings, while their presence in standard complete blood count and biochemical panels ensures that no additional testing burden is placed on clinicians or patients. Furthermore, the biological relevance of these parameters to the pathophysiological processes underlying AMI, such as inflammation, thrombosis, and tissue hypoxia, strengthens their clinical credibility. Because these markers are already embedded in routine medical workflows, their utilization within predictive models minimizes implementation barriers and enhances real-world feasibility.

Building on this premise, the present study investigates the diagnostic utility of routine hematological parameters in AMI using an inherently interpretable approach grounded in XAI. Unlike traditional black-box algorithms, XAI methodologies prioritize transparency and accountability, key requirements for clinical deployment, especially in high-stakes conditions such as myocardial infarction. To this end, we employed an ENN, a class of intrinsically interpretable models that embeds interpretability directly into the model architecture. This approach allows each prediction to be not only accurate but also traceable to specific hematological features, thereby reinforcing clinical trust and facilitating evidence-based decision-making. Given that the blood parameters used are cost-effective, non-invasive, and routinely collected in most healthcare systems, their incorporation into an ENN-based diagnostic framework offers a promising avenue for scalable and interpretable AMI risk assessment.

According to the modeling results of the present study, the use of routine hematological parameters within an ENN framework enabled the accurate and interpretable classification of individuals with AMI. The model demonstrated excellent diagnostic performance, with an overall accuracy of 94.1%, sensitivity of 95.7%, and specificity of 92.8%, indicating that the approach is not only robust but also clinically relevant in distinguishing between control and AMI individuals. Beyond the numerical performance metrics, the integration of SHAP values allowed for comprehensive insight into how each hematological parameter contributed to individual and global predictions. As visualized in Figure 3, NEU, WBC, RDW-CV, and MPV emerged as the most influential predictors across the entire cohort. Elevated red cell distribution width (RDW-CV) has been consistently associated with poor cardiovascular outcomes in AMI, reflecting anisocytosis and impaired erythropoiesis driven by systemic inflammation and oxidative stress. These pathophysiological processes contribute to endothelial dysfunction, plaque instability, and microvascular impairment, which are central to the progression and severity of myocardial injury. Karakas et al. [11] demonstrated that higher RDW and neutrophil-to-lymphocyte ratio (NLR) values were predictive of left ventricular dysfunction following acute myocardial ischemia, highlighting their prognostic value in assessing post-infarction cardiac performance. Similarly, mean platelet volume (MPV) serves as a marker of platelet size and reactivity, with increased MPV indicating the presence of larger, more metabolically and enzymatically active platelets. These platelets have enhanced prothrombotic potential, releasing greater amounts of procoagulant mediators, which facilitate thrombus formation and propagate coronary occlusion after plaque rupture. Reddy et al. [29] reported that elevated MPV levels were strongly associated with adverse thrombotic profiles and worse clinical outcomes in acute coronary syndromes, underscoring the role of platelet indices as both diagnostic and prognostic biomarkers. In addition, inflammatory cell ratios, particularly NLR, provide a composite measure of the balance between neutrophil-driven tissue injury and lymphocyte-mediated immune regulation. Dang et al. [30] demonstrated that elevated NLR had significant prognostic value in AMI patients, especially in those with coexisting chronic obstructive pulmonary disease (COPD), further supporting the role of systemic inflammation in worsening cardiac outcomes. Elevated WBC counts, often driven by neutrophil predominance, reflect an acute inflammatory response that exacerbates endothelial injury, promotes oxidative stress, and accelerates atherothrombosis. According to the results, the top predictive features identified—namely RDW, MPV, and the neutrophil-to-lymphocyte ratio (NLR)—are closely aligned with well-established pathophysiological mechanisms of AMI. Elevated RDW reflects anisocytosis and impaired erythropoiesis, processes often driven by systemic inflammation and oxidative stress, both of which contribute to endothelial dysfunction and plaque instability. Increased MPV indicates the presence of larger, more reactive platelets, which promote thrombus formation and propagate coronary occlusion following plaque rupture. Likewise, a high NLR represents a heightened inflammatory state characterized by neutrophil-mediated tissue injury and lymphocyte suppression, amplifying myocardial damage and impairing healing. By linking these predictive biomarkers to fundamental mechanisms such as inflammation, thrombogenesis, and myocardial hypoxia, the model’s outputs are grounded in established cardiovascular biology, enhancing both the interpretability and clinical credibility of its predictions.

Collectively, the convergence of these findings indicates that the ENN’s prioritization of inflammatory markers (NEU, WBC, NLR), red cell heterogeneity (RDW-CV), and platelet activity (MPV) is not only statistically robust but also biologically coherent with established AMI pathophysiology. This alignment between model-derived feature importance and mechanistic cardiovascular biology reinforces the interpretability, clinical credibility, and translational potential of the proposed ENN-based diagnostic approach.

This finding is consistent with previous studies that have highlighted elevated neutrophil and RDW values as being associated with worse cardiovascular outcomes and increased inflammatory burden in AMI patients [31,32].

Importantly, the study went beyond global interpretability and explored individual-level SHAP analyses, as depicted in Figure 4 and Figure 5, which presented two examples each from the control and AMI-classified subgroups. These personalized visualizations demonstrated that while some features like NEU and RDW-CV consistently contributed to classification decisions, the weight and direction of influence varied across individuals, highlighting the heterogeneous nature of AMI presentations. For example, in subject P001 (control), low neutrophil and RDW-CV values played protective roles, whereas in subject P005 (AMI), elevated NEU and WBC were strong positive contributors to the AMI classification. This individualized reasoning enhances clinical trust, as it allows practitioners to understand not just what the model predicts, but why it arrives at those predictions for each patient.

Furthermore, class-wise feature contribution comparison shown in Figure 6 revealed that certain parameters, such as WBC and NEU, had stronger predictive power in AMI cases, whereas other features like HGB (hemoglobin) or EO (eosinophil count) exhibited relatively balanced or minimal influence across classes. These inter-class differences in feature importance emphasize the added value of explainable methods in highlighting pathophysiologically distinct biomarker profiles, which may not be visible through conventional statistical comparisons alone.

Complementing this, Figure 7 presented a comparative distribution analysis of key hematological features between the control and AMI groups. These plots not only confirmed statistical differences in features like NEU, RDW-CV, and WBC, but also visually illustrated the extent of overlap and separation between groups. Such visualization supports the biological plausibility of the selected features and justifies their inclusion in the diagnostic model.

Collectively, the findings of this study demonstrate that commonly available, low-cost blood parameters can be effectively leveraged through interpretable machine learning techniques to support early and accurate diagnosis of AMI. The proposed model, based on the ENN framework, not only achieved a high level of diagnostic performance—with an overall accuracy of 94.1%, sensitivity of 95.7%, and specificity of 92.8% but also maintained full transparency in its decision-making process. Such performance is especially impressive given the simplicity and routine nature of the input variables, indicating that advanced diagnostic capabilities can be realized without the need for costly or invasive procedures.

In addition to its methodological contributions, the present study extends and advances upon the findings of a previous work that utilized the same hematological dataset for AMI prediction using traditional ensemble models such as LightGBM and SHAP-based explainability techniques [33]. While that study effectively demonstrated the diagnostic potential of hematological parameters through post-hoc XAI methods, it did not incorporate intrinsically interpretable neural network architectures. In contrast, our study employs an Explainable Neural Network (ENN) approach—specifically the Neural Additive Model (NAM)—which not only embeds interpretability within the model architecture but also achieves substantially higher diagnostic performance (accuracy: 94.1% vs. 83%). This demonstrates that interpretable models do not necessarily require a trade-off in performance and can outperform black-box methods when properly optimized for clinical data.

The integration of ENN modeling with SHAP analysis offers a dual advantage: robust predictive accuracy and a transparent, interpretable rationale behind each prediction. This transparency is crucial in clinical environments, where trust, accountability, and the ability to justify decisions are essential components of effective care delivery. Unlike conventional black-box models that often obscure the reasoning behind their outputs, the ENN architecture used in this study allows clinicians to understand exactly how and why a particular prediction was made—down to the level of individual biomarker contributions in each case.

Moreover, the model’s strong generalization across both AMI and control subjects, combined with the ability to generate case-specific interpretability profiles, demonstrates its utility not only as a diagnostic tool but also as a clinician-aligned support system. This alignment with clinical reasoning enhances its acceptability, facilitates integration into existing workflows, and reduces the risk of algorithmic bias or overfitting to spurious patterns. From a translational perspective, the use of routinely collected, non-invasive data means that the model could be readily deployed in a variety of healthcare settings, including emergency departments, primary care facilities, and even telemedicine platforms. To the best of our knowledge, this study represents the first clinical application of ENN to real-world healthcare data, marking a novel integration of this architecture in a biomedical setting. While previous ENN implementations have been largely theoretical or applied to synthetic or non-clinical datasets, our study pioneers the use of ENNs on actual clinical laboratory data, specifically routine hematological parameters for disease classification. This not only advances the field of interpretable machine learning but also provides a proof-of-concept for deploying ENNs in high-stakes medical decision-making. The successful application of an ENN in the context of AMI diagnosis demonstrates its broader feasibility and transformative potential for various clinical domains.

Furthermore, as evidenced by the comparative analysis presented in Table 3, the proposed ENN consistently outperformed even the most established and well-known black-box models, including Random Forest, SVM, and XGBoost, across nearly all primary classification metrics. By achieving the highest accuracy, balanced accuracy, F1-score, MCC, and sensitivity, the ENN demonstrated not only superior predictive capability but also maintained complete transparency in its decision-making process. This unique combination of performance and interpretability underscores the ENN’s potential to set a new benchmark for diagnostic modeling in cardiovascular medicine, offering clinicians both the diagnostic power of advanced machine learning and the trustworthiness of fully explainable predictions.

### Limitation

Despite promising results, several important limitations must be acknowledged. First, the retrospective nature of this study inherently introduces potential selection bias and limits our ability to control for confounding variables that might influence hematological parameters in AMI patients. The single-center design, while ensuring data consistency and standardized laboratory protocols, significantly restricts the generalizability of our findings to broader populations with varying demographic characteristics, comorbidity profiles, and healthcare infrastructures. The sample size, although adequate for initial model development and internal validation, may not fully represent the heterogeneous clinical presentations and laboratory variations observed across different ethnic groups, age ranges, and geographic regions.

Additionally, the study lacks external validation cohorts, which are crucial for assessing model robustness and real-world performance. The temporal aspects of hematological changes during AMI progression were not captured due to the cross-sectional nature of laboratory measurements at presentation. The dataset’s lack of precise timestamps relative to symptom onset represents a particularly significant limitation, as hematological parameters exhibit dynamic temporal changes during acute coronary events. This temporal variability could significantly impact parameter interpretation and model predictions, as blood samples obtained at different time points post-symptom onset may reflect distinct pathophysiological phases of the acute coronary syndrome. Furthermore, potential confounding factors such as pre-existing hematological disorders, concurrent medications affecting blood parameters, and timing of blood sample collection relative to symptom onset were not comprehensively controlled.

Future research should prioritize large-scale, multicenter prospective studies incorporating diverse patient populations to validate these findings. Longitudinal studies tracking hematological parameter evolution throughout the AMI course would provide valuable insights into dynamic biomarker patterns. Integration of additional clinical variables, genetic factors, and advanced machine learning techniques may further enhance model accuracy and clinical utility. These validation efforts are essential before considering implementation in routine clinical practice.

Second, while the use of an inherently interpretable model such as the ENN enhances transparency, the level of interpretability may still be challenging for non-technical clinical users without appropriate visualization tools or training. While SHAP values provide valuable insights into feature contributions, they require familiarity with machine learning concepts that may hinder widespread adoption of clinical interpretations.

While the present study focused solely on routinely collected hematological parameters, future research could explore the integration of additional clinical signals such as electrocardiographic (ECG) findings, cardiac biomarkers (e.g., troponin), and vital signs. Recent studies have demonstrated that multimodal diagnostic strategies—combining laboratory, imaging, and physiological data—are becoming standard practice in chest pain triage, leading to improved diagnostic accuracy and more timely clinical interventions. Incorporating these complementary modalities into an explainable machine learning framework may enhance predictive performance, increase generalizability across diverse patient populations, and support broader clinical applicability in real-world emergency care settings.

Finally, although some hematological variables (e.g., Hb, HCT, MCV, and MCH) are inherently correlated, the NAM assumes independent additive contributions. While the model demonstrated stable performance in cross-validation, this structural assumption remains a limitation. Future studies could employ interaction-enabled NAMs or alternative explainable models to better capture correlated feature effects. Another significant limitation of this study is the lack of a formal multicollinearity assessment. Hematological parameters naturally exhibit complex intercorrelations and multicollinearity due to their physiological and mathematical relationships. These interdependencies can be categorized into several groups. First, physiologically related parameters such as red blood cell indices (hemoglobin, hematocrit, mean corpuscular volume, and mean corpuscular hemoglobin) are both mathematically and physiologically linked, as they collectively describe red blood cell characteristics and oxygen-carrying capacity. Similarly, white blood cell parameters, including total WBC count and its differential components (NEU, LY, MO, EO, BA), show inherent dependencies since they represent constituent parts of the leukocyte population, with changes in one component naturally affecting the relative proportions of others. Second, mathematically derived indices such as NEU/LY, PLT/LY, MPV/LY, and LY/MO are inherently correlated with their constituent variables, reflecting computational rather than independent biological processes. These natural intercorrelations are intrinsic to hematological data and represent fundamental characteristics of blood parameter analysis rather than methodological flaws. Although such multicollinearity is unlikely to severely impair the overall predictive performance of the NAM—given its robustness through separate feature-specific subnetworks that independently model non-linear relationships while preserving additive interpretability—it may influence the distribution of feature importance and thus complicate clinical interpretability. The NAM’s assumption of independent additive contributions may not fully capture complex physiological dependencies, potentially leading to redistribution of importance scores among correlated features. Future studies should, therefore, include explicit multicollinearity diagnostics (e.g., variance inflation factors or correlation matrices), explore interaction-enabled NAM architectures capable of modeling feature dependencies, or apply dimensionality reduction techniques to better account for correlated predictors while maintaining clinical interpretability.

## 5. Conclusions

In conclusion, this study demonstrates that our ENN-based model, built on the NAM framework, offers a unique combination of high diagnostic accuracy and intrinsic interpretability, setting it apart from existing explainable AI approaches in the context of AMI prediction. By learning smooth, feature-specific functions, the model reveals transparent and clinically relevant relationships between hematological parameters and AMI risk, thereby minimizing the dependence on post-hoc explanation tools. When further enhanced with SHAP analysis, it achieves both global and local interpretive depth, ensuring that its predictions remain transparent, trustworthy, and clinically actionable. Beyond its technical performance, this ENN-based framework represents a scalable, ethically sound, and clinically viable decision-support system that aligns with the principles of precision medicine and responsible AI in healthcare. Its unique ability to merge interpretability with high performance positions it as a promising tool for reducing diagnostic delays and improving patient outcomes in the critical care setting of AMI.

## Figures and Tables

**Table 1:** Comparison of hematological parameters between the AMI and control groups.

Variable *	Group		*p* **
	Control	AMI	
WBC (10^3^/L)	7.5 (2.282)	10.38 (4.5)	<0.001
RBC (10^12^/L)	4.7 (0.685)	4.79 (0.82)	0.384
HGB (g/dL)	13.7 (2.125)	13.9 (2.5)	0.107
HCT (10^2^/L)	0.04 (0.04)	0.06 (0.04)	<0.001
EO (10^3^/L)	0.13 (0.13)	0.1 (0.15)	0.004
LY (10^3^/L)	2.28 (0.883)	2.36 (1.76)	0.077
MO (10^3^/L)	0.56 (0.215)	0.7 (0.39)	<0.001
NEU (10^3^/L)	4.3 (1.745)	6.45 (3.93)	<0.001
NLR	1.89 (0.912)	2.697 (2.23)	<0.001
PLR	111.462 (52.1)	102.632 (67.846)	0.002
MPVLR	4.32 (1.832)	3.866 (3.273)	<0.001
SWR	4.13 (1.735)	3.526 (2.466)	<0.001
MPV (fL)	10.1 (1.3)	9.4 (1.69)	<0.001
PLT (10^3^/L)	256.5 (84.5)	253 (92)	0.499
PDW (%)	41 (5.625)	41.2 (6.41)	0.265
MCV (fL)	87.2 (5.325)	86.3 (6.6)	0.003
RDW-SD	41 (4.425)	41.2 (4.7)	0.117
RDW-CV	13.2 (1.3)	13.6 (1.3)	<0.001
MCH (pg)	29.1 (2.4)	29.3 (2.8)	0.095
MCHC (g/dL)	33.2 (1.9)	33.9 (1.6)	<0.001
PDW (fL)	12 (3.625)	15.9 (5.29)	<0.001
PCT (%)	0.255 (0.07)	0.23 (0.09)	<0.001

*: Variables are presented as median (IQR), **: Mann–Whitney U test was used.

**Table 2:** Diagnostic performance indicators for the ENN with 95% confidence intervals.

Performance Metrics	Value	95% CI Lower Limit	95% CI Upper Limit
Accuracy	0.941	0.918	0.965
Balanced Accuracy	0.942	0.919	0.965
F1-Score	0.939	0.915	0.963
MCC	0.883	0.852	0.915
Sensitivity	0.957	0.917	0.981
Specificity	0.928	0.884	0.959
Positive Predictive Value	0.922	0.874	0.956
Negative Predictive Value	0.96	0.923	0.983
Positive Likelihood Ratio	13.267	8.14	21.622
Negative Likelihood Ratio	0.047	0.024	0.092

**Table 3:** Comparative performance results with 95% bootstrap confidence.

Model	Accuracy	Balanced Accuracy	F1-Score	MCC	Sensitivity	Specificity	PPV	NPV	ROC AUC
Random Forest	0.906 (0.878, 0.935)	0.905 (0.875, 0.935)	0.899 (0.866, 0.933)	0.814 (0.766, 0.860)	0.870 (0.829, 0.911)	0.940 (0.907, 0.974)	0.930 (0.893, 0.968)	0.887 (0.852, 0.922)	0.974 (0.953, 0.997)
SVM	0.875 (0.847, 0.904)	0.873 (0.844, 0.903)	0.863 (0.830, 0.896)	0.753 (0.707, 0.800)	0.817 (0.776, 0.858)	0.929 (0.896, 0.963)	0.914 (0.877, 0.952)	0.847 (0.811, 0.880)	0.942 (0.920, 0.964)
XGBoost	0.929 (0.902, 0.957)	0.928 (0.899, 0.959)	0.925 (0.892, 0.958)	0.858 (0.811, 0.905)	0.911 (0.870, 0.952)	0.946 (0.913, 0.979)	0.939 (0.902, 0.977)	0.921 (0.885, 0.956)	0.983 (0.960, 1.000)
ENN	0.941 (0.918, 0.965)	0.942 (0.919, 0.965)	0.939 (0.915, 0.963)	0.883 (0.852, 0.8915)	0.957 (0.917, 0.981)	0.928 (0.884, 0.959)	0.922 (0.874, 0.9956)	0.960 (0.923, 0.983)	0.980 (0.966, 1.000)

**Table 4:** Modeling results for STEMI and NSTEMI groups.

Performance Metric	Value
Doğruluk (Accuracy)	0.9100
Dengeli Doğruluk (Balanced Accuracy)	0.9100
Matthews Korelasyon Katsayısı (MCC)	0.8199
Kesinlik (Precision)	0.9082
Duyarlılık (Recall/Sensitivity)	0.9082
F1 Skoru (F1-Score)	0.9082
ROC Eğrisi Altındaki Alan (ROC-AUC)	0.9776

**Table 5:** NAM and SHAP variable importance value.

Feature	NAM Importance	SHAP Importance
WBC	0.188	0.240
NEU/LY	0.138	0.168
PLT	0.118	0.120
NEU	0.108	0.115
EO	0.093	0.082
LY/MO	0.088	0.080
Gender (0: female, 1: male)	0.076	0.065
RDW-SD	0.070	0.053
PDW	0.066	0.048
MCH	0.062	0.042

## Data Availability

The data presented in this study are available in Heart disease at https://doi.org/10.17632/m482gb564t.1.

## Abbreviations

The following abbreviations are used in this manuscript:

AMI	Acute Myocardial Infarction
SHAP	SHapley Additive exPlanations
ENN	Explainable Neural Network
NAM	Neural Additive Model
STEMI	ST-segment elevation MI
NSTEMI	non-ST- segment elevation MI
